# Breaking the clean room barrier: exploring low-cost alternatives for microfluidic devices

**DOI:** 10.3389/fbioe.2023.1176557

**Published:** 2023-04-27

**Authors:** Cristian F. Rodríguez, Valentina Andrade-Pérez, María Camila Vargas, Andrés Mantilla-Orozco, Johann F. Osma, Luis H. Reyes, Juan C. Cruz

**Affiliations:** ^1^ Department of Biomedical Engineering, Universidad de Los Andes, Bogotá, Colombia; ^2^ Department of Chemical and Food Engineering, Universidad de Los Andes, Bogotá, Colombia

**Keywords:** microfluidics, lab-on-a-chip, organ-on-a-chip, microfabrication, biochips, chip materials

## Abstract

Microfluidics is an interdisciplinary field that encompasses both science and engineering, which aims to design and fabricate devices capable of manipulating extremely low volumes of fluids on a microscale level. The central objective of microfluidics is to provide high precision and accuracy while using minimal reagents and equipment. The benefits of this approach include greater control over experimental conditions, faster analysis, and improved experimental reproducibility. Microfluidic devices, also known as labs-on-a-chip (LOCs), have emerged as potential instruments for optimizing operations and decreasing costs in various of industries, including pharmaceutical, medical, food, and cosmetics. However, the high price of conventional prototypes for LOCs devices, generated in clean room facilities, has increased the demand for inexpensive alternatives. Polymers, paper, and hydrogels are some of the materials that can be utilized to create the inexpensive microfluidic devices covered in this article. In addition, we highlighted different manufacturing techniques, such as soft lithography, laser plotting, and 3D printing, that are suitable for creating LOCs. The selection of materials and fabrication techniques will depend on the specific requirements and applications of each individual LOC. This article aims to provide a comprehensive overview of the numerous alternatives for the development of low-cost LOCs to service industries such as pharmaceuticals, chemicals, food, and biomedicine.

## 1 Introduction

Labs-on-a-chip (LOCs), also known as microfluidic devices, have revolutionized biomedical and chemical analysis by providing efficient, portable, and highly miniaturized solutions (Najjar et al., 2022; Izadifar et al., 2023; Zhao et al., 2023). The microfluidic design of these devices enables precise control of fluid flows and processes, leading to improved accuracy and repeatability of results (Karthik et al., 2022; Verma & Pandya, 2022). However, their high material costs and specific manufacturing processes, which need cleanroom settings, make them inaccessible to many laboratories (Pandey et al., 2017; Walsh et al., 2017).

Silicon and glass are the most common materials utilized in the manufacture of microfluidic devices (Wang Y et al., 2021; Yagyu et al., 2022). Silicon is a popular choice due to its chemical compatibility, resistance, and design flexibility (da Ponte et al., 2021). While glass is transparent, biocompatible, and electrically insulating (Orazi et al., 2022; Shubhava et al., 2022). However, both materials are expensive to manufacture and require specialized equipment and cleanroom facilities to ensure their purity and reliability (Elvira et al., 2022; Leung et al., 2022).

To overcome these obstacles, this review presents a complete analysis of different materials and methods that can be employed to build low-cost LOCs. The aim of this review is to make the benefits of microfluidic devices accessible to a broader range of researchers and to encourage the field’s continued development. Alternatives to silicon and glass will be presented, including alternative materials and fabrication techniques that can be used to produce microfluidic devices at a reasonable cost (Campbell et al., 2021; Niculescu et al., 2021; Scott & Ali, 2021).

## 2 Materials

### 2.1 Paper (cellulose and cellulose derivatives)

Paper is a cellulosic material that has become increasingly popular for fabricating microfluidic devices due to its advantageous properties (Martinez et al., 2007; Tsagkaris et al., 2021; Zhuang et al., 2022). Its distinct characteristics, such as low cost and wide availability, make it an attractive option for mass production. (Gao B et al., 2019; Kumawat et al., 2022; Zhu Y et al., 2022). There are numerous fabrication techniques available for paper, such as laser cutting, flexographic printing, screen printing, wax-based printing, and alkyl ketene dimer (AKD) printing, which make it viable and cost effective (Olmos et al., 2019; Parween et al., 2020; Ardakani et al., 2022; Lin et al., 2022; Fiore et al., 2023).

Paper’s hydrophilicity enables fluid manipulation without pumps, and its ability to be functionalized provides additional advantages (Yetisen et al., 2013). Recent advances in surface modification techniques, such as molecular imprinting, electrochemistry, or immunoassay, have allowed precise modification of paper’s surface chemical properties, resulting in precise fluid flow control and enhanced functionality (Cate et al., 2015; Boonkaew et al., 2021; Zhu L et al., 2022; Wang et al., 2023). These advances have led to the development of point-of-care tests with integrated detection functionalities that exhibit fluorescent, electrochemical, and microfluidic colorimetric paper chips (Cincotto et al., 2019; Ma et al., 2020; Tran et al., 2021). However, paper’s weak mechanical strength restricts its application in high-pressure environments, and its vulnerability to degradation limits its use in harsh conditions (Yetisen et al., 2013; Carrell et al., 2019). Moreover, irregular porosity in the paper can cause inaccuracies in regulating fluid flow, leading to reduced precision in the obtained results Nishat et al., 2021; Qin et al., 2021). To address these challenges, there has been a recent trend in using synthetic microfluidic paper composed of polymers. This innovative material offers several advantages over traditional paper, including improved consistency, more favorable surface chemistry, predictable pore size, and greater control over physical properties (Hansson et al., 2016; Zhou et al., 2021).

In line with this trend, Lin et al. (2022) have developed a novel and cost-effective technique for creating microfluidic paper-based analytical devices (μPADs). These μPADs can perform multiplexed enzyme-linked immunosorbent assays (ELISAs) for the detection of two cancer biomarkers, namely, alpha fetoprotein (AFP) and carcinoembryonic antigen (CEA). The fabrication process of these μPADs involves using nitrocellulose (NC) membranes as a substrate and polyurethane acrylate (PUA) as a barrier material to define flow channels and reaction zones. The PUA is applied to the NC membrane using screen-printing and then cured under UV light to form a hydrophobic barrier. This process allows the formation of precise and consistent channels, which are essential for accurate fluid flow regulation and the detection of the cancer biomarkers (Lin et al., 2022).

### 2.2 Hydrogels

Hydrogels are a versatile class of materials composed of cross-linked polymeric chains that have the remarkable ability to store a significant amount of water (Sharma & Tiwari, 2020; Bento et al., 2023; Tevlek et al., 2023). One of their most notable characteristics is their high degree of porosity, which makes them highly responsive to external stimuli such as temperature, pH, and ionic strength (García-Torres et al., 2022; Camman et al., 2023). Numerous hydrogels possess a notably high permeability, facilitating cell proliferation. Additionally, these hydrogels exhibit optical transparency, allowing visualization of internal microfluidic device components. These features render hydrogels particularly well-suited for use in lab-on-a-chip and organs-on-a-chip systems, especially in the context of biosensing and drug delivery applications (Hou et al., 2017; Nie et al., 2020; Ma et al., 2022; Sood et al., 2022; Cao et al., 2023).

Hydrogels can be made from natural, synthetic, or hybrid materials, depending on the specific application (Mahinroosta et al., 2018; Ho et al., 2022). Natural materials such as gelatin, chitosan, silk, and collagen provide better biocompatibility, biodegradability, and mimicry of the extracellular matrix. However, their cross-linking behavior can limit their functionality (Kato et al., 2021; Vera et al., 2021; Zhou et al., 2022). In contrast, synthetic materials such as polyethylene glycol, polyvinyl alcohol, poly (n-isopropyl acrylamide), and poly (hydroxyethyl methacrylate) have superior mechanical strength and shape memory but lack biocompatibility (Bashir et al., 2020; Nie et al., 2020; Vera et al., 2021). Ongoing research focuses on developing new hydrogel materials and improving their properties to address existing limitations and enable their use in more complex applications (Park et al., 2021; Clancy et al., 2022). In line with this rationale, Grebenyuk et al. (2023) have designed a hydrogel for precise microvessel printing at scales below the diffusion limit of living tissues, enabling the culture of large-scale engineered tissues *in vitro* over long periods, while avoiding hypoxia or necrosis. The formulation uses a photopolymer based on polyethylene glycol diacrylate (PEGDA), which incorporates the photocrosslinker pentaerythritol triacrylate (PETA) to increase the polymer’s density via crosslinking. Additionally, Triton-X 100 is used as an inert filler to maintain sufficient porosity for rapid molecule diffusion (Grebenyuk et al., 2023).

### 2.3 Polydimethylsiloxane (PDMS)

Polydimethylsiloxane (PDMS) is the most commonly used elastomer due to its compatibility with biological samples, optical clarity, and ease of fabrication for valves and pumps (de Almeida Monteiro Melo Ferraz et al., 2020; Dabaghi et al., 2021; Yandrapalli et al., 2021; Crevillen et al., 2022; Mistretta et al., 2022; Natsuhara et al., 2022). However, one of the major drawbacks of PDMS is its tendency to swell in the presence of certain solvents, particularly hydrocarbons, which can causee device failure (Raj & Chakraborty, 2020; Miranda et al., 2021). Additionally, PDMS has adsorptive properties for certain molecules that can affect the accuracy of experiments or their analyses (Akther et al., 2020; Grant et al., 2021). Water evaporation through the channel walls is a concern because it can lead to alterations in solution concentration, negatively affecting experimental results (Schneider et al., 2021b; Xia et al., 2022). The hydrophobicity of PDMS can make it difficult to fill microfluidic channels with aqueous solutions, resulting in fluid flow and analyte detection difficulties (Akther et al., 2020; Hu H et al., 2020).

### 2.4 Thermoplastics

In recent years, there has been a noticeable trend towards utilizing thermoplastic materials for fabricating microfluidic devices. This trend has gained traction due to the distinct advantages of thermoplastics, including lower costs and faster production times, rendering them a viable option for scaling up production (Becker & Gärtner, 2000; Tsao, 2016). Among the polymers that have gained significant usage in microfluidics are Polycarbonate (PC), Polyethylene terephthalate (PET), Polyimide (PI), Polypropylene (PP), Polystyrene (PS), Cyclic olefin copolymers (COC), and Poly (methyl methacrylate) (PMMA) (Denecke et al., 2022; Lee et al., 2022; Ortegón et al., 2022; Persson et al., 2022; Matsuura & Takata, 2023; Thaweeskulchai & Schulte, 2023).

Polystyrene (PS) is a highly stable and non-polar linear polymer that contains benzene as a pendant group (Bhavsar et al., 2018; Shakeri et al., 2022a). Its exceptional properties, including high mechanical and thermal stability, chemical resistance, biocompatibility, and optical transparency, make it an attractive material for microfluidic device fabrication (de Oliveira et al., 2021; Zhou et al., 2021). Furthermore, PS substrates can have their optical properties adjusted by incorporating various dopants in their structure, making it possible to tailor the optical properties of PS substrates to suit specific applications in microfluidics (Bhavsar et al., 2018; Sivakumar & Lee, 2020). However, it is important to note that PS can undergo degradation or physical and chemical changes upon exposure to light of wavelengths below 380 nm (Bhavsar et al., 2018).

Cyclic olefin copolymers (COC) in particular, are a type of thermoplastic material composed of cyclic olefin monomers and linear olefins (Bruijns et al., 2020; Agha et al., 2022). COC possesses numerous advantageous properties, including low water absorption, excellent electrical insulation, long-term surface treatment stability, and resistance to a broad range of acids and solvents (Shakeri et al., 2022b; Guan et al., 2022). It is an ideal material for use in diverse applications, such as biological, membrane, and semiconductor fields (Etxeberria et al., 2022; Xia et al., 2023). COC is remarkable for its stiffness, optical clarity, and heat deflection temperature, which varies between 70°C and 170°C, depending on the COC grade (Bruijns et al., 2019; Agha et al., 2022). Microchannels in COC can be fabricated using a variety of processes, including micromilling, injection molding, and heat embossing (Gang et al., 2019; Shakeri et al., 2022b; Li et al., 2022; Qin Y et al., 2022).

PMMA, in particular, is an inexpensive thermoplastic polymer with excellent optical properties, such as high transparency and refractive index (Kulsharova et al., 2018; Vo & Chen, 2022). Because it is easy to manipulate, it is a suitable material for the fabrication of microfluidic devices (Horowitz et al., 2020; Persson et al., 2022; Madadi et al., 2023; Zolti et al., 2023). PMMA is also biocompatible and can be sterilized easily by various sterilization methods, such as autoclaving (Guzzi et al., 2020; Khot et al., 2020) and chemical sterilization (Nguyen et al., 2019; Ameri et al., 2022), making it useful for biotechnological and biomedical applications. However, PMMA has some limitations, including high hydrophobicity and low mechanical strength (Amirabad et al., 2022; Shakeri et al., 2022a; Kulkarni & Goel, 2022).

The trend of utilizing polymers for microfluidic device fabrication is evident in the pursuit of novel materials that can be effectively employed in microfluidic devices. For example, Kaya et al. (2022) have introduced programmable polymer magnetic composites that incorporate droplets of solid-liquid phase change material, each containing a single magnetic dipole particle. These composites can be reprogrammed into four different states: superparamagnetic, artificial spin ice, spin glass, and ferromagnetic. Also, they possess high remanence characteristics along with Curie temperatures below the composite’s degradation temperature (Kaya et al., 2022). The development of such innovative materials offers new tools that can be utilized in microfluidics to develop advanced devices and enhance the design of labs-on-a-chip.

### 2.5 Surface functionalization techniques

Various surface functionalization techniques have been developed to enhance the properties of polymers used in microfluidics devices, such as PDMS and PMMA (Shakeri et al., 2021). Plasma treatment is among these techniques, which has been shown to improve the wettability and adhesion to other materials (Sui et al., 2021; Gizer et al., 2023). In addition, techniques like chemical vapor deposition (CVD) and graft polymer coating can improve the surface’s chemical and mechanical stability (Dabaghi et al., 2019; Fan et al., 2019). Furthermore, protein adsorption and layer-by-layer (LBL) deposition teachniques can produce a biologically active surface and a multilayered coating, thereby enhancing the functionality of the devices (Babaei et al., 2022; Siddique et al., 2021; Z. Li et al., 2021). These techniques have significantly improved the properties of surfaces and expanded their potential applications in various fields, including biomedical research and point-of-care diagnostics (Y. He et al., 2022; Khemthongcharoen et al., 2021; Tang et al., 2021).

## 3 Manufacturing techniques

Conventional manufacturing techniques used for microfluidic devices require a clean room and generate high costs, which hinder their widespread adoption (Walsh et al., 2017; Shakeri et al., 2021). Consequently, there has been a growing interest in developing low-cost techniques to fabricate microfluidic devices, facilitating broader access and expediting integration into various applications (Li et al., 2022; Akhtar et al., 2023; Li et al., 2023).

In general, two types of techniques can be used to manufacture microfluidic devices at a low cost: subtractive and additive techniques (Niculescu et al., 2021; Scott & Ali, 2021; Ching et al., 2023). In subtractive techniques, material is removed from a substrate to create microchannels and other features, whereas in additive techniques, material is added layer by layer to build a three-dimensional structure (Bhatia & Sehgal, 2021; Kulkarni, Salve, et al., 2021; Mesquita et al., 2022). Common subtractive techniques include hot embossing and laser ablation, while common additive techniques include 3D printing, soft lithography, and inkjet printing (Bavendiek et al., 2020; Schneider et al., 2021a; Grebenyuk et al., 2023).

These techniques offer various advantages, including low-cost, rapid prototyping, and the ability to manufacture microfluidic devices with complex geometries (Chen et al., 2020; Santana et al., 2020). However, there are limitations to these methods that must be considered. This contribution will examine the advantages and disadvantages of these low-cost techniques.

### 3.1 Soft lithography

Soft lithography is a highly popular technique for developing microfluidic devices, which utilizes soft and elastomeric materials such as polydimethylsiloxane (PDMS) to create microfluidic patterns (Whitesides et al., 2001; Phiphattanaphiphop et al., 2020; Duc et al., 2021; Šakalys et al., 2021).

**FIGURE 1:**
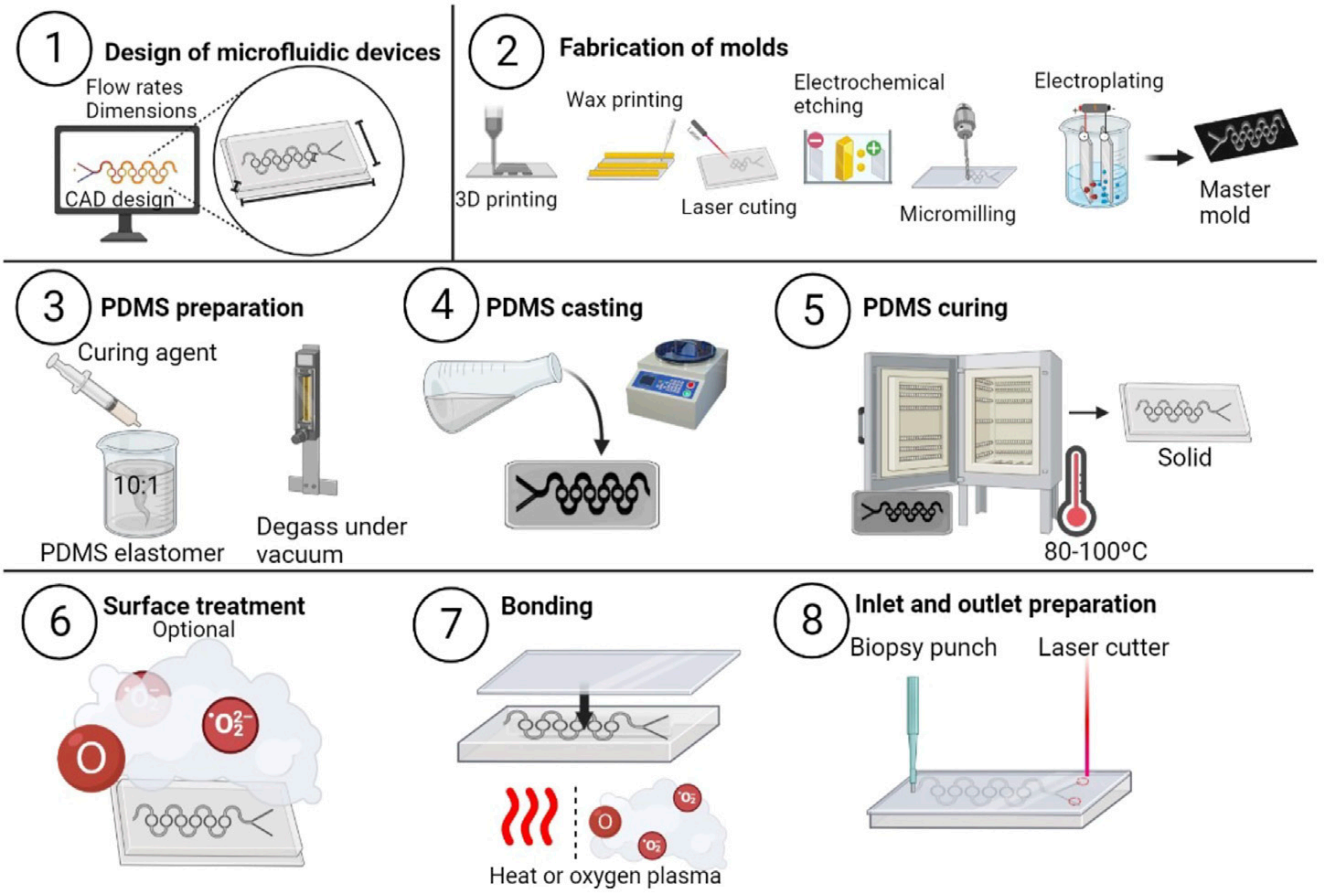
Soft lithography method to produce microfluidic devices. The procedure consists of the steps listed below: 1) Using CAD software, design the microfluidic device in consideration of the device’s specifications. 2) Create the master mold using a variety of methods, such as 3D printing, wax printing, laser cutting, micro milling, or electrochemical etching. 3) Create the PDMS solution by combining the PDM and curing agent with the PDMS. 4) Using a spin coater or by hand, evenly coat the mold with the PDMS mixture. 5) Cure the PDMS layer in an oven at temperatures between 80°C and 100°C for a few hours. The PDMS should then be removed from the mold and sized and shaped as desired. 6) Treat the surface of the PDMS device. Attach the PDMS device to the substrate’s lid using oxygen plasma or a thermal bonding technique. Utilize a biopsy punch or a laser cutter to create the required inlets and outlets in the PDMS.

Figure 1 illustrates the key phases of the soft lithography method, beginning with the design of the microfluidic device using computer-aided design (CAD) software (Bressan et al., 2019; Sommonte et al., 2022). Subsequently, a patterned substrate known as the “master” is created using expensive techniques, which offer excellent resolution. These techniques include photolithography (250 nm), nanoimprint lithography (15 nm), x-ray lithography (15 nm), or electron beam lithography (10 nm), but are primarily used in large-scale manufacturing facilities (Maldonado & Peckerar, 2016; Gale et al., 2018; Thuau et al., 2018; Matsumoto et al., 2020; Phiphattanaphiphop et al., 2022). Consequently, there has been a growing interest in employing low-cost manufacturing techniques, including micromilling (25 μm), 3D printing (5–100 μm), and laser cutting (25 μm) (Gale et al., 2018; Rusling, 2018; Hamilton et al., 2021; Šakalys et al., 2021; Preetam et al., 2022; Qin S et al., 2022).

After producing the master, a mixture of PDMS elastomer and curing agent is prepared and poured into the mold (Kamei et al., 2015; Mukherjee et al., 2019). The mixture is spread evenly using a spin coater or by hand and then baked between 75°C and 150°C for several hours until it solidifies (Jang et al., 2016; B. Parker et al., 2016). The cured PDMS is then removed from the mold and cut to the desired dimensions and shape. To ensure excellent adhesion and wetting of the PMDS, the receiving substrate is treated with a surface modifier, such as oxygen plasma treatment, UV/ozone treatment, chemical modification, or physical abrasion (Long et al., 2017; Borók et al., 2021; Mondal et al., 2021; Darboui et al., 2022).

Finally, inlets and outlets are cut into the PDMS using a biopsy punch or laser cutter (Mamidanna et al., 2017; Lee et al., 2018). Soft lithography is a promising and cost-effective method for fabricating microfluidic devices, making it a valuable tool for researchers and small-scale manufacturing facilities (Heo et al., 2020; Šakalys et al., 2021; Nguyen et al., 2022).

### 3.2 Laser ablation

Due to their precise material removal capabilities, lasers have gained significant interest in recent years for producing microfluidic devices (Ghosh et al., 2019; Mansour et al., 2022; Wei et al., 2023). Laser energy can be focused on the substrate to initiate thermal degradation by optically amplifying the light (Ravi-Kumar et al., 2019; Ho et al., 2022). The laser pulses break polymer bonds, causing an increase in temperature and the expulsion of the material (Vargas et al., 2019; Ouyang et al., 2022). The wavelength of the laser is crucial for determining the quality of the resulting microfluidic devices (Roth et al., 2018; Scott & Ali, 2021) and can be categorized into two types: long or ultra-short wavelengths (Wang et al., 2017; Niculescu et al., 2021). Long-wavelength lasers can achieve widths up to 100 μm (Sarma & Joshi, 2020), while ultra-short wavelength lasers can produce channels with widths as small as 10 μm (Meineke et al., 2016; Roth et al., 2019).

**FIGURE 2:**
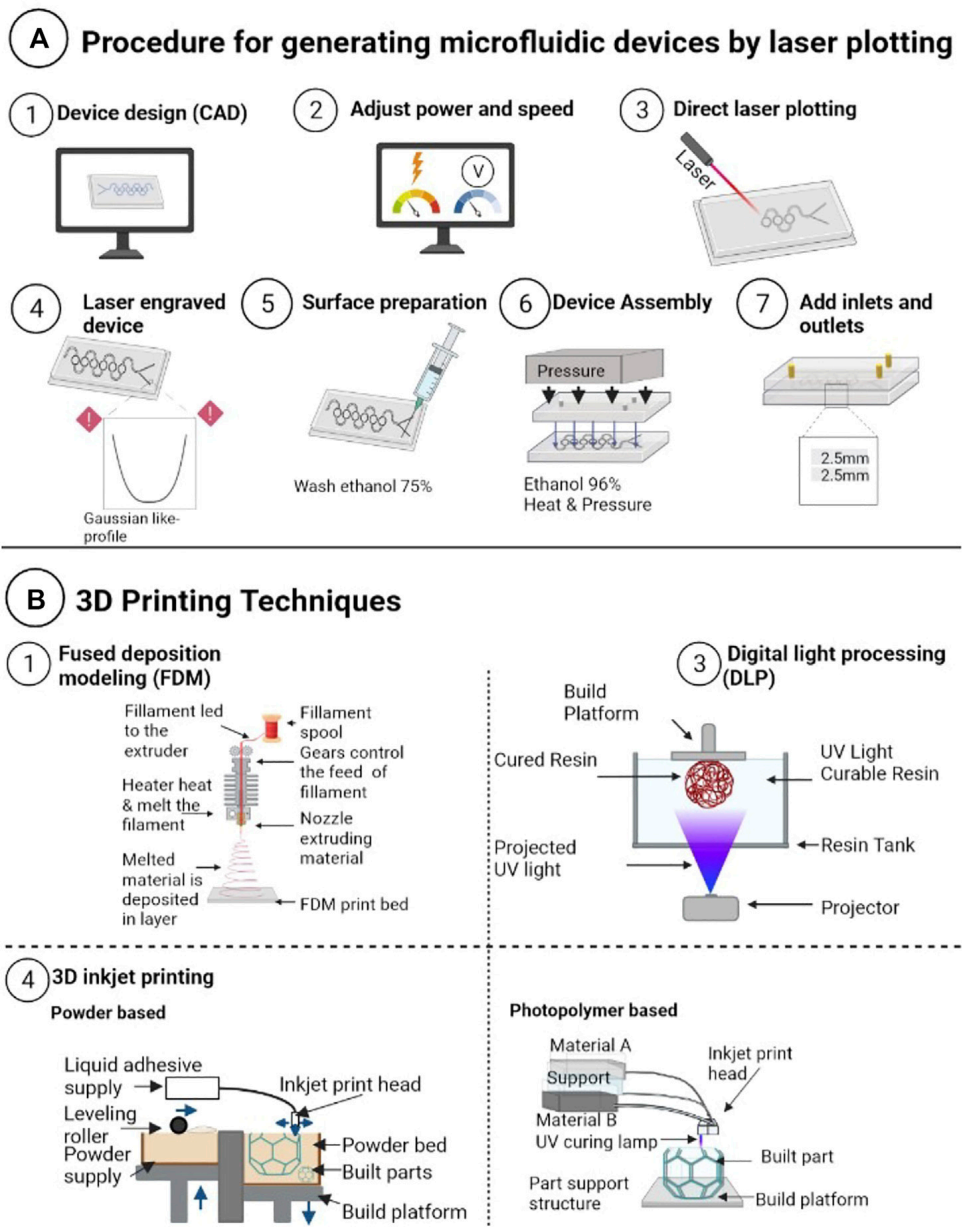
**(A)**: 3D printing techniques adapted from Prabhakar et al. (2021), and Padash et al. (2020), including: 1) Fused Deposition Modeling (FDM), which involves heating and extruding material through a nozzle to create layers of the microfluidic device. 2) Stereolithography (SLA) uses a UV laser to solidify resin and generate the device. 3)Digital Light Processing (DLP), which selectively shines light onto the resin using a digital projector. **(B)**: Laser engraving process for creating a microfluidic device, which includes the following steps: 1) CAD software is used to design the device. 2) Adjust the laser’s speed and power to achieve the desired microchannel depth. 3) Using laser engraving, which has the disadvantage of producing a Gaussian profile. 4) To remove residues, wash the surface with 75% ethanol. 5) Using 96% ethanol and heat, join the device’s lid to the bottom layer. 6) Gluing the device’s inputs and outputs.

As depicted in Figure 2, laser ablation is a multistep process. The microfluidic device is initially designed using CAD software (Isiksacan et al., 2016; Guo et al., 2021). The design is then used to control the movement of the laser, which removes substrate material. The laser can remove material in a variety of patterns, including straight lines, curved lines, and complex shapes (Wlodarczyk et al., 2019; Hu X et al., 2020). The depth of engraving is determined by the laser’s speed and power, both controlled by software (Hubeatir et al., 2018; Mansour et al., 2022). The laser moves back and forth over the substrate to create the desired microfluidic channels by removing material in a controlled manner (Yao & Fan, 2021; Katla et al., 2023).

Laser ablation offers several advantages, including its low-cost and rapid manufacturing process, which can be applied to various materials, including polymers, glass, metals, and ceramics (Malecha et al., 2019; Remiszewska et al., 2019; Wlodarczyk et al., 2019). Polymers such as PMMA are frequently utilized due to their thermal stability and versatility. However, as confirmed by microscopy techniques, this method has the disadvantage of causing a Gaussian degradation, resulting in diagonal walls with a grainy texture in the channel walls (Figure 2) (Matellan & del Río Hernández, 2018; Gao K et al., 2019; Guo et al., 2022).

### 3.3 3D printing

In recent years, 3D printing has gained popularity as a method for fabricating microfluidic devices due to its capacity to create precise, intricate structures (Markoski et al., 2021; Shan et al., 2021; Wang A et al., 2021; Wang H et al., 2021). This method offers a wide range of material options, including metals, ceramics, and polymers such as PDMS, polycarbonate (PC), PMMA, and acrylonitrile butadiene styrene (ABS) (Litti et al., 2021; Abdalkader et al., 2022; Fei et al., 2022; Li et al., 2023).

Numerous 3D printing techniques exist, such as Selective Laser Sintering (SLS), Electron Beam Melting (EBM), stereolithography (SLA), and Multi Jet Fusion (MJF) (Roy et al., 2019; Berger et al., 2021; Hwang et al., 2022; Uçak et al., 2022). However, due to the high equipment and material costs, these methods may not be suitable for smaller-scale production and research facilities with limited resources (Mehta & Rath, 2021; Griffin & Pappas, 2023). As a result, alternative low-cost techniques have been developed to enable the 3D printing of microfluidic devices, such as Fused Deposition Modeling (FDM), Digital Light Processing (DLP), and Inkjet 3D printing (i3Dp), (Kanitthamniyom et al., 2021; Vasilescu et al., 2021; Duarte et al., 2022). These techniques provide the advantages of 3D printing, such as design flexibility and production speed, without the high costs of more advanced methods (Au et al., 2016; Prabhakar et al., 2021).

Inkjet 3D printing (i3Dp) is an exceptionally precise additive manufacturing technique that utilizes a printhead to deposit photocrosslinkable resin onto a build platform in a layer-by-layer process, resulting in a continuous pattern (Mehta & Rath, 2021; Gonzalez et al., 2022). This process is versatile, efficient, and exceptionally precise, with a resolution of around 10 μm (Zhou et al., 2020; Mehta & Rath, 2021; Gonzalez et al., 2022; Su et al., 2023). It is important to note that i3DP has two main types: powder-based and photopolymer-based. In powder-based i3Dp, a polymeric sticking solution is used to agglomerate powder particles, while the photopolymer-based i3Dp deposits small droplets of both the build and support materials in a layer-by-layer process to fabricate an object (Padash et al., 2020; Wang & Chen, 2020; Prabhakar et al., 2021).

FDM is the most popular low-cost technique for manufacturing microfluidic devices due to its affordability (Ballacchino et al., 2021; Mader et al., 2021; Mehta et al., 2021). This method has a resolution around 100 μm (Zhou et al., 2020). It creates microfluidic devices by heating and extruding thermoplastic polymeric materials through a nozzle and depositing successive layers onto a cooled surface in an X-Y plane (Lynh & Pin-Chuan, 2018; Zhang et al., 2020). Numerous researchers favor it due to its relative simplicity, user-friendliness, and quicker production time (Gaal et al., 2017; Bauer & Kulinsky, 2018; Quero et al., 2022).

DLP is a 3D printing method that uses photopolymerization to create microfluidic devices with a resolution of around 5 μm (Amoyav et al., 2020; van der Linden et al., 2020; Zhou et al., 2020; Bucciarelli et al., 2022). It employs a digital projector to selectively shine light on the resin, allowing for faster printing because an entire layer of resin can be exposed at once (Catterton et al., 2021; Tabriz et al., 2022). Due to the high pixel density of the digital projector, this method typically has a higher resolution; however, some other 3D printing technologies produce smoother and more accurate surfaces due to the precision of the laser beam (Rey-Joly Maura et al., 2021; Musgrove et al., 2022; Chen et al., 2023).

DLP printers are distinguished by their remarkable efficiency and cost-effectiveness when compared to similar methods. Notably, they are considerably more affordable than Stereolithography (SLA) techniques, which often require expensive equipment and materials (Tully & Meloni, 2020; Yoo et al., 2021). However, the use of a single resin in the production of microfluidic devices presents a significant challenge in achieving varying material properties across different components of the device. Grayscale digital light processing (g-DLP) has emerged as a promising technique to overcome this limitation. A prime example of this has been demonstrated by Yue et al. (2023), who have developed a photocurable resin using g-DLP that offers high stretchability and a broad range of modulus values. By utilizing three different monomers and incorporating aliphatic urethane diacrylate (AUD) as a crosslinker, the resin can form hydrogen bonds with both isobornyl acrylate (IBOA) and 2-hydroxyethyl acrylate (2-HEA) to achieve moduli values ranging from 0.016 to 478 MPa, with a stretchability of up to 1,500% in its soft state (Yue et al., 2023).

The choice of 3D printing technique to produce microfluidic devices is contingent on the application and available resources. Factors such as cost, resolution, and surface accuracy must be considered when selecting the most appropriate technique (Waheed et al., 2016; Mehta & Rath, 2021; Gonzalez et al., 2022).

## 4 Discussion

Labs-on-a-chip (LOCs) devices have been made possible by microfluidic technology, which enables the miniaturization and optimization of processes. The industry has been focusing on their development (Mistretta et al., 2022; Najjar et al., 2022). However, traditional techniques for producing LOCs are expensive, limiting their availability and creating a need for more efficient and cost-effective alternatives (Ren et al., 2013; Elvira et al., 2022). Consequently, several low-cost microfluidic device manufacturing techniques have emerged to replace traditional manufacturing techniques requiring clean room facilities (Leung et al., 2022; Mistretta et al., 2022).

This article compares soft lithography, laser plotting, and 3D printing, three low-cost techniques for manufacturing microfluidic devices. Soft lithography is an affordable technique that offers high resolution and versatile geometries (Leung et al., 2022; Mesquita et al., 2022). Laser plotting is a rapid method (Scott & Ali, 2021; Šakalys et al., 2021), while 3D printing provides design flexibility and fast production (Niculescu et al., 2021; Griffin & Pappas, 2023).

In addition, the review examines the suitability of diverse materials, including paper, polymers, and hydrogels, for producing low-cost LOCs. Paper microfluidics devices are inexpensive, user-friendly, and portable but have limitations in mechanical strength and reusability (Cate et al., 2015; Prabhakar et al., 2021). Because of their high compatibility with biological samples and ease of fabrication, PDMS and PMMA are frequently employed in microfluidic device production. However, they have drawbacks, such as PDMS’s tendency to swell when exposed to certain organic solvents and PMMA’s low mechanical strength. Hydrogels have modifiable mechanical properties and can mimic the extracellular matrix (ECM), but their mechanical strength and long-term stability are limited (Hsu et al., 2018; Qin et al., 2021).

The material chosen for the device is determined by its specific requirements and intended application, as each material has its own advantages and disadvantages. The development of low-cost manufacturing techniques for LOCs will increase their availability and accelerate their incorporation into various applications. This review offers an overview of the potential routes to enhance LOC accessibility for a wider range of researchers and support the ongoing growth of microfluidics.
